# EFHD1: A Potential Prognostic Biomarker Related to Mitochondrial Function and Aging in Atherosclerosis Plaque

**DOI:** 10.2174/0109298673356904250628182630

**Published:** 2025-07-21

**Authors:** Lin Wang, Yuxiu Han, Yu Qiao, Tao Yan, Zhi Qi, Wei Zhang, Ling Xin, Mingjing Yu, Zhili Chen

**Affiliations:** 1 Key Laboratory of Post-Neurotrauma, Neurorepair, and Regeneration in Central Nervous System, Ministry of Education and Tianjin City, Department of Neurology, Tianjin Neurological Institute, Tianjin Medical University General Hospital, Tianjin, 300052, China;; 2 Department of Neurology, Tianjin Hospital, Tianjin University, Tianjin, 300211, China

**Keywords:** Atherosclerosis, aging, mitochondrial dysfunction, EFHD1, GEO, calcium signaling pathway

## Abstract

**Introduction:**

Atherosclerosis (AS) is prevalent among the elderly population and poses a significant global health burden. However, the precise underlying mechanisms linking aging and mitochondrial dysfunction in AS remain unclear.

**Methods:**

Through comprehensive utilization of databases including the Gene Expression Omnibus (GEO), MitoCarta, Molecular Signatures Database (MSigDB), and Human Aging Genomic Resources (HAGR), we employed various bioinformatics methods to explore the possible function of EF-hand domain family member D1 (EFHD1). This included the functional enrichment analysis, immune cell infiltration, and the lncRNA-miRNA-EFHD1 network. The validity of EFHD1 was confirmed using additional datasets and through Receiver Operating Characteristic (ROC) curve evaluation. Lastly, *in vitro* experiments were conducted using THP-1 cells treated with oxidized low-density lipoprotein (ox-LDL) to validate the expression and function of EFHD1 through Western blot and real-time quantitative PCR analyses. Additionally, *in vivo* experiments were performed on ApoE^-/-^ mice exhibiting atherosclerotic phenotypes, utilizing immunofluorescence staining.

**Results:**

Totally seven genes associated with aging and mitochondrial function (ALDH3A2, UCP1, BCL2, EFHD1, AHCYL1, HTRA2, and ALDH9A1) were discovered in AS, with EFHD1 identified as the principal hub gene. Immune infiltration analysis indicated that EFHD1 was negatively associated with myeloid suppressor cells (MDSC), activated B cells, and natural killer cells. An evident decline in EFHD1 was noted in unstable or advanced plaques compared to stable or early plaques, accompanied by significant area under the ROC curve (AUC) values of 0.917 (GSE100927) and 0.933 (GSE41571). Moreover, we recorded a reduction in EFHD1 expression in AS tissues and macrophages treated with ox-LDL. Following the silencing of EFHD1, TNF-α and IL-1β decreased, while ALODA, PKM2, MMP-9, JAK2, and STAT3 levels were upregulated. Furthermore, levels of ATP and reactive oxygen species (ROS) were diminished, while calcium ions and mitochondria levels remained unchanged.

**Discussion:**

To date, the common pathogenic genes associated with aging and mitochondrial dysfunction in atherosclerotic disease have been scarcely investigated. Using bioinformatics approaches, we identified seven hub genes (ALDH3A2, UCP1, BCL2, EFHD1, AHCYL1, HTRA2, and ALDH9A1) related to mitochondrial function and aging. Among these, EFHD1 was determined as the final hub gene. As a calcium sensor, EFHD1 plays a pivotal role in regulating mitochondrial metabolism and has been implicated in the prognosis of various tumors. Our findings demonstrated that EFHD1 knockdown decreased the levels of pro-inflammatory cytokines, such as IL-1β and TNF-α, increased JAK2 and STAT3 protein levels, and elevated MMP-9 levels, all of which may contribute to the vulnerability and progression of atherosclerotic plaques.

**Conclusion:**

Our research revealed a reduction in EFHD1 expression within atherosclerotic tissues, suggesting its potential role in inflammation and mitochondrial energy metabolism as a key regulator of the calcium signaling pathway. This discovery offers possible advancements in the early diagnosis and treatment strategies for AS.

## 1 INTRODUCTION

The rupture of atherosclerotic plaques can lead to thrombosis and a reduction in the vascular lumen, obstructing blood flow and precipitating a range of cardiovascular and cerebrovascular diseases. Gaining insight into the causes and mechanisms behind atherosclerosis (AS) is vital.

The increasing global population of seniors, especially individuals aged 65 and older, positions AS as a leading cause of illness and death in this demographic. In addition to hypertension and dyslipidemia, vascular aging has emerged as an independent driver of AS [1, 2]. Vascular aging is characterized by a gradual decline in arterial elasticity, leading to increased stiffness of blood vessels [3]. Furthermore, research indicates that blood vessels in older individuals exhibit elevated levels of several pro-inflammatory molecules [4] and an enhanced uptake of plasma lipoproteins. In the elderly population, various cell types, including vascular smooth muscle cells [5], endothelial cells [6], and inflammatory cells,display characteristics consistent with cellular senescence. Senescent endothelial and smooth muscle cells exhibit telomere attrition, with an average of 30% shortening of AS plaques compared to healthy vessels [7]; mitochondrial reactive oxygen species (ROS) overproduction, showing a 2.5-fold increase in aged macrophages [8]; and senescence-associated secretory phenotype (SASP)-mediated inflammation, where increased IL-6 and IL-8 levels are associated with plaque instability [9]. This well-established paradigm positions AS as a catalyst for accelerating vascular aging. However, there are few studies on aging-related genes in AS. A recent investigation found aging-related CCAAT/enhancer-binding protein beta (CEBPB) related to anti-inflammatory polarization in foam cell formation [10]. Additionally, CAV1, Nox4, NINJ1, YAP1, and ABI3 have been known to be cellular senescence-associated genes that contribute to the progression of atherogenesis [11].

Mitochondria serve as the energy centers of human cells, primarily responsible for the production of adenosine triphosphate (ATP) [12]. Cellular metabolism revolves around the production and consumption of ATP. Mitochondrial dysfunction contributes to atherosclerosis (AS) through two primary pathways: bioenergetic collapse and epigenetic metabolic reprogramming. In terms of bioenergetic collapse, ATP synthesis in senescent foam cells decreases by more than 50% [13], impairing effective cellular function. Regarding epigenetic metabolic reprogramming, mitochondrial DNA (mtDNA) oxidation, indicated by an 8-hydroxydeoxyguanosine (8-OHdG) level with a plaque burden correlation of r=0.72 [14], activates the Janus kinase 2 (JAK2) and signal transducer and activator of transcription 3 (STAT3) signaling pathways, thereby promoting macrophage senescence.

Meanwhile, a reduction in the quality and function of mitochondria is linked to the natural process of aging and is associated with the onset of several diseases related to age [15]. With advancing age, the integrity, volume, and functionality of mitochondrial DNA diminish because of the accumulated oxidative damage and mutations caused by ROS. Mitochondria may be influenced by normal aging, which in turn regulates cellular senescence and age-related diseases. Identifying the common genes linking mitochondrial function and cellular senescence may provide valuable insights for tailoring treatment strategies for AS. However, currently, there is a lack of adequate evidence regarding the relationship between senescence-associated genes and mitochondrial genes in the AS process. Nonetheless, a more profound understanding of common genes related to mitochondrial function and aging is vital for formulating more effective therapeutic approaches aimed at alleviating the burden of AS.

In this study, we discovered 852 aging-related genes that are differentially expressed (DEGs), sourced from the GSE111782 dataset. By utilizing the MitoCarta 3.0 database, we retrieved a total of 1,136 genes. Furthermore, 979 aging-related genes were aggregated from the Molecular Signatures Database (MSigDB) and the Human Aging Genomic Resources (HAGR) database. Subsequently, EF-hand domain family member D1(EFHD1) was identified as the key gene through Weighted Gene Co-expression Network Analysis (WGCNA) and Receiver Operating Characteristic (ROC). Ultimately, we validated the expression of EFHD1 in both an atherosclerosis animal model and a foam cell model. To further explore the function of EFHD1 in macrophages, we measured inflammatory mediators like IL-1β and TNF-α, as well as indicators related to mitochondrial metabolism, including intracellular calcium, ATP, ROS, and mitochondrial function in THP-1 cells after knocking down EFHD1. This study marks the first attempt to incorporate DEGs associated with mitochondrial activity and aging within the context of atherosclerosis. By elucidating these molecular pathways, we seek to offer fresh perspectives on the formation of atherosclerotic plaques and emphasize new potential treatment options.

## 2 MATERIALS AND METHODS

### 2.1 Dataset Collection

A total of eight AS-associated datasets were sourced from the Gene Expression Omnibus (GEO) database, which included GSE111782 [16], GSE28829 [17], GSE104140 [18], GSE100927 [19], GSE41571 [20], GSE163154 [21], GSE43292 [22], and GSE120521 [23]. The GSE111782 dataset comprised 9 asymptomatic carotid plaque samples and 9 symptomatic carotid plaque samples, while GSE28829 included 13 samples of early atherosclerotic plaques alongside 16 samples of advanced atherosclerotic plaques. In GSE104140, there were 13 samples of early atherosclerotic plaques alongside 19 samples of advanced atherosclerotic plaques, whereas GSE100927 encompassed 12 control samples and 29 carotid atherosclerosis samples. The GSE41571 dataset had 6 stable plaque samples and 6 ruptured plaque samples, while GSE163154 included 27 intraplaque hemorrhage(IPH) samples and 16 non-IPH samples. GSE43292 featured an equal number of 32 samples of early and advanced atherosclerotic plaques, and GSE120521 had 4 samples each of stable and unstable plaques.

### 2.2 Access to Mitochondria-associated Genes

The MitoCarta3.0 database was utilized to gather mitochondria-associated genes [24]. This database employs advanced mass spectrometry techniques to analyze mitochondria isolated from 14 different tissues. It assesses protein localization using large-scale green fluorescent protein (GFP) tagging combined with microscopy and integrates these findings with the Bayesian algorithm alongside six other genomic datasets about mitochondrial localization. It includes 1,136 genes in humans and 1,140 genes in mice that are associated with mitochondria.

### 2.3 Aging and Senescence-associated Genes

Totally 979 senescence and aging-related genes were derived from the MSigDB gene sets and HAGR [25], including GOBP cellular senescence (MM10157), GOBP aging (MM5100), and GOBP multicellular organism aging (MM5399), GOBP replicative senescence (MM10158), Jiang aging cerebral cortex down (MM702), Jiang aging cerebral cortex up (MM650), Jiang aging hypothalamus down (MM656), Jiang aging hypothalamus up (MM703), Reactome DNA damage telomere stress-induced senescence (MM14904), and Reactome cellular senescence (MM14901) [26, 27].

### 2.4 Identification of DEGs

The online analysis tool GEO2R [28] is based on Limma and GEOquery packages in R software. In this study, GEO2R was applied to analyze and compare gene expression profiles between symptomatic and asymptomatic carotid plaques in the GSE111782 dataset. Differential gene expression analysis identified genes that met the criteria of a |log2FC (fold change)| of 0.5 or greater, and an adjusted *P*-value below 0.05 as DEGs [29]. Volcano plots were created through the ggplot2 package. To pinpoint common DEGs related to mitochondria and aging, we used an online tool for Venn diagrams (https://bioinformatics.psb.ugent.be/webtools/Venn/).

### 2.5 Analysis of Weighted Gene Correlation Network

The WGCNA was conducted through the 'WGCNA' package of R software [30] in this research. This methodology organizes genes into distinct modules while examining the connections between these modules and various disease traits. The genes, exhibiting the highest 25% variance from the GSE28829 and GSE104140 datasets, were used to develop the co-expression network. The dynamic cutting tree technique was utilized to consolidate the modules with a threshold of 0.25. Additional criteria for constructing the co-expression network included determining the soft threshold power (β) based on the scale-free topology standard (with an independence index of R^2^=0.9) [31], utilizing the pickSoftThreshold function, and ensuring that every module contained a minimum of 30 genes. To assess the relationships between modules and different stages of AS plaque progression, Pearson correlation analysis was utilized.

### 2.6 Validation of Critical Gene Signatures

The ROC curves were utilized to assess the diagnostic relevance of AS-related signatures, and the area under the ROC curve (AUC) was calculated by utilizing the 'pROC' package in R software [32]. A statistical significance threshold of *p* < 0.05 was set for two-tailed tests. The accuracy of key biomarkers was further validated using the datasets GSE163154, GSE43292, GSE104140, GSE28829, GSE120521, and GSE41571 to evaluate gene signature expression in AS plaques. Additionally, multivariate analysis using R software was conducted to determine the association between candidate genes and clinical factors.

### 2.7 Gene Functional Enrichment Analysis

A heatmap highlighting the most differentially expressed genes based on EFHD1 expression was created, followed by gene set enrichment analysis (GSEA). The 'clusterProfiler' package in R software [33] was utilized to conduct Gene Ontology (GO) and Kyoto Encyclopedia of Genes and Genomes (KEGG) pathway analyses, aiming to examine the signaling pathways and biological processes in which genes related to EFHD1 in AS plaques may be involved.

### 2.8 Construction of the lncRNA-miRNA-EFHD1 Network and Chemical Network

MicroRNAs (miRNAs) and long non-coding RNAs (lncRNAs) are important in modulating gene expression at both transcriptional and posttranscriptional levels. To investigate the factors involved in regulating important gene signatures, we utilized tools such as miRNet [34], ENCORI and starBase [35] to predict lncRNAs, and miRDB [36], miRWalk [37], miRabel [38], and TargetScan [39] to predict miRNAs associated with DEGs. The Comparative Toxicogenomics Database (CTD) [40] was employed to identify compounds linked to these genes. Regulatory networks were analyzed and visualized through the Cytoscape software [41].

### 2.9 Analysis of Immune Infiltration

To compare the immune infiltration status of symptomatic versus asymptomatic carotid plaques, we calculated the standardized enrichment scores through single-sample gene set enrichment analysis (ssGSEA). The enrichment scores for 28 immune cells [42] from endometrium samples were quantified using gene signatures and the R software package 'GSVA' [43]. A heat map was generated to illustrate immune infiltration within the samples using the 'pheatmap' package. Subsequently, the 'ggstatsplot' package was utilized for Spearman correlation analysis involving immune cells and diagnostic markers, with visualization of the results done using the 'ggplot2' package. Additionally, a correlation heatmap showcasing the relationships among immune-associated genes was produced with the 'corrplot' package.

### 2.10 Cell Culture

THP-1 cells were procured from Procell Company located in Wuhan, which were cultured in Roswell Park Memorial Institute (RPMI) 1640 medium (G4535, Servicebio, Wuhan, China), enriched with 10% fetal bovine serum (FBS), along with 100 U/mL penicillin and 100 μg/mL streptomycin (P1400, Solarbio, Beijing, China), in an incubator at a controlled temperature of 37°C with 5% CO _ 2 _ atmosphere. For experiments, a six-well cell culture plate was used to maintain the cells. To induce differentiation into THP-1 macrophages, Phorbol 12-myristate 13-acetate (200 ng/mL, PMA, MedChemExpress, USA) was introduced to THP-1 monocytes for 48 hours [44]. Subsequently, we used either 50 μg/mL or 80 μg/mL of oxidized low-density lipoprotein (oxLDL) (Yiyuan Biotech, Guangzhou, China) for an additional 48 hours to establish the foam cell model [45].

### 2.11 Transfection of Small Interfering RNA (siRNA)

THP-1 cells were transfected using RFect^SP^ V2 siRNA Transfection Reagent (Cat#11049, Baidai Biotechnology, Changzhou, China) mixed with 50 nM siRNA against EFHD1, based on the manufacturer's instructions. Following transfection, the cells were maintained for another 48 or 72 hours. The efficiency of gene knockdown was evaluated through western blot analysis. SiRNA targeting EFHD1 and the corresponding negative control RNAs were synthesized chemically by Ribobio (Guangzhou, China). The antisense sequence of siEFHD1 is 5’-GGCCAGTAAGTTTGAAGCA-3’.

### 2.12 Measurement of Intracellular Calcium, ATP, ROS, and Mitochondria in THP-1 Cells

Cells were treated with 2 μM Fura-2 AM (S1052, Beyotime, Beijing, China) at 37°C for 30 minutes to determine intracellular calcium contents by scanning with a microplate reader after washing [46]. Intracellular levels of ROS were evaluated with a Reactive Oxygen Species Assay Kit (S0033M, Beyotime, Beijing, China) [47]. ATP production in THP-1 cells was determined using the ATP Detection Assay (KTB1019, Abbkine, Wuhan, China). Additionally, 200 nM Mito tracker dye (C1049B, Beyotime, Beijing, China) was added to THP-1 cells, followed by a 30-minute incubation period at 37°C [48]. The stained mitochondria were observed *via* fluorescence microscopy.

### 2.13 Western Blot Analysis

Tissues or cells were lysed using RIPA and PMSF (Solarbio, Beijing, China). The concentration of protein was assessed with the BCA protein assay kit (Solarbio, Beijing, China). Then, 20 μg proteins were segregated through 10% SDS-PAGE gel electrophoresis and transferred to a PVDF membrane. This membrane was incubated overnight with primary antibodies targeting JAK2 (1:1000, 3230, CST, USA), STAT3 (1:1000, 9139, CST, USA), EFHD1 (1:1000, H00080303-M05, Abnova, Taiwan, China), Pyruvate kinase M2 isoform (PKM2) (1:1000, 15822-1-AP, Proteintech), Aldolase A (ALDOA) (1:1000, 11217-1-AP, Proteintech, Wuhan, China), Matrix Metalloproteinase-9 (MMP-9) (1:1000, ab38898, Abcam, UK), and β-actin (1:1000, AC026, ABclonal, Wuhan, China) at 4°C. After three washes using Tris-buffered saline with Tween-20 (TBST) for 5 minutes each, the membranes were then treated with the suitable secondary antibody against mouse or rabbit IgG (1:5000, Signalway, Maryland, USA) for 1 hour at room temperature. The antibody signals were detected with ECL detection reagent (GlpBio, USA), and the bands were analyzed with ImageJ software.

### 2.14 Real-time Reverse Transcription-polymerase Chain Reaction (RT-PCR)

Total RNA was isolated from THP-1 cells with Trizol reagent (GlpBio, USA), and cDNA synthesis was conducted with a reverse transcription kit (GenStar, Beijing, China). Quantitative PCR was conducted using a QuantStudioTM 3 real-time PCR instrument (ThermoFisher, USA) and 2X SYBR Green Fast qPCR Mix kit (ABclonal, Wuhan, China). Normalization of results was done against Gapdh control values. The relative expression levels of genes were determined employing the 2^-ΔΔCT^ method. The primer sequences utilized were: for EFHD1, 5′-TTTCGAGTTTGCGAGGAGCGCGTC-3′ (forward) and 5′-CATAACGACGAATCGCAAAACGCG-3′ (reverse); for TNF-α, 5′-CCTCTCTCTAATCAGCCCTCTG-3′ (forward) and 5′-GAGGACCTGGGAGTAGATGAG-3′ (reverse); for IL-1β, 5′-ATGATGGCTTATTACAGTGGCAA-3′ (forward) and 5′-GTCGGAGATTCGTAGCTGGA-3′ (reverse); for Gapdh, 5′-CTGGGCTACACTGAGCACC-3′ (forward) and 5′-AAGTGGTCGTTGAGGGCAATG-3′ (reverse).

### 2.15 Animal Models

Seven male 8 week old wild-type (C57BL/6) and 24 apolipoprotein E knockout (ApoE−/−) mice, each weighing approximately 25 ± 2 g [49-52], were sourced from the Vital River Corporation located in Beijing, China. Mice were kept in SPF-grade facilities maintained at a temperature between 22 and 24°C, with 45% humidity and a 12 hour light/dark routine. The mice had free access to food and water during the study period. Various groups of mice were randomly allocated to specific treatment regimens. For 16 weeks, the control group was provided a standard diet (1022, hfkbio, Beijing, China), while the AS group was given a high-fat diet (HFD) (D12108C, 40% fat, 1.25% cholesterol, xiaoshuyoutai, Beijing, China) for either 12 or 16 weeks [53] to promote the development of early or advanced atherosclerotic plaques. Following treatment, aortic tissues were harvested, and the plasma was collected to assess the gene expression and atherosclerotic lesions. The presence of lipid deposition in the arterial tissue was confirmed as a successful AS model through pathological staining analysis. All experiments were conducted following the US National Research Council's “Guide for the Care and Use of Laboratory Animals”. All animal experiments were performed following the ARRIVE guidelines.

### 2.16 Body Weight Measurement and Lipid Assay

Body weights were measured at baseline and on weeks 4, 8, 12, and 16 after feeding a high-fat diet (HFD) using automatic devices. Blood samples were obtained *in vivo*, with plasma extracted through centrifugation at 3000 rpm for 10 minutes at 4°C. Plasma lipid levels, such as total cholesterol (TC) and low-density lipoprotein cholesterol (LDL-C), were evaluated using kits from Shanghai MLBIO Biotechnology Co., Ltd.

### 2.17 Evaluation of Atherosclerotic Lesions

After inhaling 1.5% isoflurane for 2 min to induce anaesthesia, mouse aortas were longitudinally opened from the heart to the iliac arteries. Tissue samples were isolated and preserved in 4% paraformaldehyde (P1110, Solarbio, Beijing, China) at 4°C for 24 hours. Some samples were embedded in paraffin (P100931, Aladdin, Shanghai, China), sectioned at 8 μm, and analyzed for aorta morphological histomorphometric characteristics using hematoxylin/eosin (H&E) staining (G1120, Solarbio, Beijing, China). Other samples were processed by embedding in optimal cutting temperature (OCT) medium (4583, SAKURA, USA), followed by sectioning at 15 μm and stained through Oil Red O (G1262, Solarbio, Beijing, China) to visualize atherosclerotic plaques [52, 54]. Captured images were taken with a light microscope (Nikon, Japan).

### 2.18 Immunofluorescence Staining

Immunofluorescence staining of atherosclerotic lesions was conducted on 8 µm sections of aortas that were freshly embedded in OCT. The sections were initially treated with antibodies targeting CD68 (1:200, 53969, Signalway, Maryland, USA) and EFDH1 (1:100, 250045, ZenBio, Chengdu, China) at 4°C overnight. Subsequently, the sections were incubated with secondary antibodies (1:500, A-11035/A32723, Invitrogen, Waltham, MA, USA) for 1 hour. Following a wash with PBS, DAPI (ab104139, Abcam, UK) was utilized to stain the cell nuclei, and a fluorescence microscope was used to capture images.

### 2.19 Statistical Analysis

The statistical analyses were performed with R software (version 4.2.2). Data was statistically analyzed with GraphPad Prism 9.5 software and expressed as mean ± standard deviation. To compare two groups with one variance, an unpaired t-test was applied, while for multiple comparisons, a one-way ANOVA test was employed. A *p*-value below 0.05 was deemed statistically significant, indicated by ****p < 0.0001, ***p < 0.001, **p < 0.01, *p < 0.05, with 'ns' signifying no significance.

## 3 RESULTS

### 3.1 Identification of DEGs Related to Mitochondrial Function and Aging in AS

The dataset GSE111782 underwent normalization and processing, as illustrated in Fig. (**1A**). Analysis of the volcano plot revealed 852 DEGs in the GSE111782 dataset, satisfying the conditions of adjusted *p*-value below 0.05 and |log2FC|≥0.5 (Fig. **1B**), consisting of 390 upregulated genes and 462 downregulated genes. Subsequently, 1136 genes related to mitochondrial dynamics in humans were identified from the MitoCarta 3.0 database. In addition, 979 genes related to aging and senescence were retrieved from the HAGR database and the MSigDB gene sets. Intersecting by the Venn diagram, 7 common genes, aldehyde dehydrogenase 3 family member A2 (ALDH3A2), uncoupling protein 1 (UCP1), BCL2, EFHD1, adenosylhomocysteinase like 1 (AHCYL1), high-temperature requirement protease A2 (HTRA2), and aldehyde dehydrogenase 9 family member A1 (ALDH9A1) were identified as common DEGs associated with mitochondrial function and aging in AS (Fig. **1C**).

### 3.2 WGCNA Analysis Revealed EFHD1 as the Hub Gene in Symptomatic Carotid Plaques

To investigate the highly correlated genes among the intersecting mitochondrial and aging-related genes in various stages of AS plaque progression, WCGNA was conducted. Initially, a cutoff R^2^ value of 0.9 was applied to select an appropriate soft threshold β value for the GSE28829 and GSE104140 datasets, ensuring that the constructed networks mimic real biological networks following a power law distribution. Subsequently, we performed a hierarchical clustering analysis utilizing weighted correlation, leading to the segmentation of clustering outcomes into gene modules according to predefined criteria. The minimum size of the modules was 30, and genes exhibiting similar expression patterns were eventually grouped into a total of 13 modules (Figs. **2A**, **B**). Within the GSE28829 datasets, the turquoise module exhibited the most significant negative correlation (ME = -0.682, *p* = 4.6e−05), while the pink, green, and yellow modules displayed strong positive associations with AS progression (Fig. **2C**). Conversely, in the GSE104140 dataset, the magenta, turquoise, and pink modules demonstrated positive correlations with plaque staging, while the brown and black modules were negatively associated with AS progression (Fig. **2D**). Subsequently, 1039 genes from GSE28829 and 979 genes from GSE104140 were identified. We used the Venn diagram to determine the core genes, which overlapped between the modules and the 7 differentially expressed genes (DEGs) mentioned earlier. Through this process, EFHD1 was found as the final hub gene.

### 3.3 Validation of EFHD1 in the GEO Database

To assess the diagnostic performance of EFHD1, a ROC curve analysis was performed, yielding an AUC value greater than 0.7. By external datasets validation, the AUC value was 0.917 (*p* < 0.001) in the GSE100927 dataset, while in the GSE41571 dataset, the AUC value was 0.933 (*p* = 0.018) (Fig. **3A**). These findings suggested that EFHD1 holds promise for predicting the development of AS plaques. Subsequently, the expression of EFHD1 was further validated through the analysis of GSE163154, GSE43292, GSE104140, GSE28829, GSE120521, and GSE41571. The findings demonstrated that EFHD1 was significantly downregulated in unstable or advanced plaques compared to stable or early plaques (Fig. **3B**). Furthermore, multivariate analysis confirmed a negative association between EFHD1 expression and the rupture or worsening of AS plaques (Table **1**).

### 3.4 GSEA, GO, and KEGG Functional Analysis of EFHD1 Gene

GSEA was carried out to explore the possible functions of the EFHD1 gene. Following regrouping based on EFHD1 expression levels, 378 DEGs were determined in dataset GSE111782 (Fig. **4A**). GSEA results indicated that the group with high EFHD1 expression was significantly enriched for infection by pathogenic *Escherichia coli*, the regulation of the actin cytoskeleton, and the contraction of vascular smooth muscle (Figs. **4B**, **C**). To further investigate the pathways and functions linked to the 378 DEGs, subsequent analyses using KEGG pathway enrichment and GO analysis were employed. The GO analysis identified enrichment in various processes, such as kinase and protein kinase activity regulation, actin filament-based process, and myeloid cell differentiation (Fig. **4D**). Regarding KEGG pathways, the top four significantly enriched pathways were the proteoglycans in tumors, contraction of vascular smooth muscle, adrenergic signaling pathway in cardiomyocytes, and cAMP signal pathway (Fig. **4E**). The results suggested that EFHD1 could be significant in regulating cellular senescence, growth, differentiation, and metabolism.

### 3.5 Construction of the lncRNA-miRNA-EFHD1 Network, and Chemical Network of EFHD1

To investigate the regulation of EFHD1, we initially focused on upstream regulation by examining microRNAs and lncRNAs targeting EFHD1. MiRNAs were identified through four online databases: TargetScan,miRDB, miRabel, and miRWalk, and 35 regulatory miRNAs were identified (Fig. **5A**). Subsequently, we explored lncRNAs interacting with these regulatory miRNAs through databases such as miRNet, ENCORI, and starBase, and constructed the lncRNA-miRNA-EFHD1 regulatory network (Fig. **5B**). Furthermore, we screened 104 compounds in the Comparative Toxicogenomics Database to identify potential therapeutic drugs based on EFHD1 gene features (Fig. **5C**). For instance, Benzo(a)pyrene, Valproic Acid, and Genistein were found to increase EFHD1 expression, while bisphenol A, Coumestrol, and Tetrachlorodibenzodioxin decreased EFHD1 expression. The establishment of the competitive endogenous RNAs (ceRNA) network and chemical network could elucidate the pathogenesis and potential therapeutic strategies for atherosclerosis.

### 3.6 Infiltration of Immune Cells and its Correlations with Key Genes

We conducted an ssGSEA analysis to evaluate the infiltration patterns of 28 distinct types of immune cells within AS plaques. Analysis of samples from GSE111782 showed the standardized enrichment score of immune infiltration (Fig. **5D**). Pearson correlation analysis revealed a negative relationship between the EFHD1 and various immune cells, like myeloid suppressor cells (MDSC), activated B cells, and natural killer(NK) cells (*p* < 0.01, Fig. **5E**). Furthermore, the relationship of immune-associated genes such as IL-11, CXCL1, CD24, MIF, STAT3, and ILF2 was presented in Fig. (**5F**). Notably, STAT3 demonstrated a robust association with EFHD1(r =0.8, *p* < 0.001).

### 3.7 EFHD1 may Affect the JAK2/STAT3 Pathway and Regulate the Function of oxLDL-treated THP-1 Cells

AS is characterized by chronic inflammation induced by the accumulation of LDL, suggesting that the regulation of inflammation-related pathways may be effective in stroke prevention. Among the genes associated with the immune response, STAT3 was selected for further investigation due to its strong connection to the JAK-STAT signaling pathway. Additionally, prior research has shown that the JAK2/STAT3 pathway influences the regulation of the AS process [18]. Consequently, the impact of EFHD1 knockdown on the JAK2/STAT3 signaling cascade was evaluated using a cell model. THP-1 cells were cultured *in vitro* and continuously stimulated with 80 μg/mL ox-LDL for 24 hours to induce foam cell formation, characterized by irregular morphology, enlarged size, and protrusions around the cytomembrane (Fig. **6A**). The lipid accumulation was confirmed through Oil Red O staining (Fig. **6B**). In comparison to the control group, the ox-LDL-treated group showed lower levels of mRNA and protein for EFHD1 in the THP-1 cell line (Figs. **6D**-**E**, Fig. **S1**). Following siRNA transfection into THP-1 cells to knock down EFHD1 (Fig. **6C**), a significant reduction in EFHD1 protein expression was observed (*p* < 0.05) (Fig. **6F**, Fig. **S1**). Knockdown of EFHD1 also reduced relative mRNA levels of pro-inflammatory cytokines, including IL-1β and TNF-α (Fig. **6G**). Further investigation into the role of EFHD1 revealed that both ox-LDL stimulation and EFHD1 knockdown decreased ATP production (Fig. **6H**). Interestingly, while si-EFHD1 reduced ROS levels, mitochondrial and calcium ion levels remained unchanged (Figs. **7A**, **B**). In the EFHD1 knockdown group, protein levels of ALODA, PKM2, MMP-9, JAK2, and STAT3 were upregulated (*P* < 0.05) (Figs. **7C**, **D**, Fig. **S1**).

### 3.8 The Validation of EFHD1 Expression in the AS Animal Model

Protein expression of EFHD1 was assessed in mice atherosclerotic tissues. In contrast to the control group, the AS mice group exhibited a marked rise in average weight from the 8^th^ week onwards (*P* < 0.01) (Fig. **8A**). Additionally, the circulating TC and LDL-C levels in the AS mice demonstrated a notable elevation (*P* < 0.001) (Fig. **8B**). By the end of the 16th week, pathological staining (Oil Red O) of mouse aortas indicated a substantial accumulation of lipid plaques, leading to lumen stenosis (Figs. **8C**, **D**). Furthermore, HE staining revealed the formation of aortic plaques in HFD-feeding ApoE^-/-^ mice (Fig. **8E**). The immunofluorescence analysis indicated a notable reduction in the EFHD1 expression within the aortic tissue and macrophages of the AS mice model group (Figs. **9A**-**D**). Moreover, results from the western blotting substantiated the diminished levels of EFHD1 in the AS mice (Figs. **9E**, **F**, Fig. **S1**).

## 4 DISCUSSION

Atherosclerosis is recognized as an inflammatory disease of the vessel wall characterized by autoimmune and dysfunctional properties [55]. Notably, aging is a major independent risk factor that accelerates the progression of atherosclerosis [3]. Mitochondrial dysfunction acts both as a contributor to and a consequence of cellular senescence, significantly affecting various feedback mechanisms that initiate and sustain the senescent phenotype [56]. Despite the acknowledged importance of aging and mitochondrial dysfunction, their interaction with age-related diseases remains insufficiently understood. Therefore, this research aims to investigate the common characteristics and molecular pathways of mitochondrial metabolism and aging-related genes in AS through a bioinformatics approach, utilizing existing sequencing databases to provide novel insights into the underlying pathogenesis.

This study is the first to investigate the common genes associated with mitochondrial function and aging in AS plaques. Through a comprehensive bioinformatics analysis of the GSE111782 dataset, we identified seven genes that are differentially co-expressed: ALDH3A2, UCP1, BCL2, EFHD1, AHCYL1, HTRA2, and ALDH9A. The BCL2 BCL2 is part of a family of proteins named after its original member, BCL2 itself. This family comprises approximately 20 proteins that play diverse roles in either promoting or inhibiting apoptosis, thereby significantly influencing the regulation of this process [57, 58]. Research conducted by Urszula Rykaczewska revealed that BCLAF1 (BCL2 [B-cell lymphoma 2]-associated transcription factor 1) functions in conjunction with BCL2 to regulate cell survival [59]. Furthermore, the silencing of BCLAF1 resulted in a decrease in BCL2 expression, leading to the formation of larger, lipid-rich necrotic cores and more susceptible plaques [60]. Thus, BCL2 may represent a crucial therapeutic target for the treatment of atherosclerosis [61]. Mitochondria function as the primary site for ATP synthesis and serve as the principal source of cellular reactive oxygen species (ROS). In this context, mitochondrial uncoupling proteins (UCPs) decrease the efficiency of oxidative phosphorylation and play a significant role in regulating mitochondrial ROS generation [60]. Substantial evidence indicates that UCP2 and UCP3, homologous to UCP1 and located in cardiac tissue, help protect against oxidative damage in mitochondria by reducing ROS production [62]. Studies have demonstrated that mitochondrial uncoupling protein 1 (UCP1) serves as a regulatory element, inhibiting the activation of the NLRP3 inflammasome and the synthesis of IL-1β in vascular tissues [63]. UCP1 likely confers protective advantages against the development of atherosclerosis. High-temperature requirement protein A2 (HtrA2/Omi) plays a critical role in the regulation of mitochondrial quality control. This protein not only facilitates the degradation of improperly accumulated proteins during the mitochondrial unfolded protein response (UPRmt) to maintain normal mitochondrial function through its enzymatic activity, but it also modulates the interactions of proteins involved in mitochondrial dynamics, thereby preserving mitochondrial morphology [64]. Furthermore, recent research has highlighted the role of HtrA2/Omi as a novel mediator in inflammation, contributing to the regulation of the inflammatory response [65]. The absence of HtrA2 may reduce the expression of pro-inflammatory cytokines in macrophages. Interestingly, a novel inhibitor of HtrA2 has demonstrated protective effects against cerebral ischemia/reperfusion injury in mice by modulating proteins involved in Fas-mediated apoptosis [66]. HtrA2/Omi proteins may influence the remodeling of macrophage polarization by affecting the reprogramming of macrophage energy metabolism. This modulation occurs through the regulation of inflammatory signaling pathways and the maintenance of mitochondrial quality, thereby contributing to the advancement and evolution of atherosclerotic plaques. Furthermore, miR-26 has emerged as a promising biomarker for atherosclerosis, capable of repressing the expression of ACC1/2, ACLY, ACSL3/4, and ALDH3A2 [67]. Nonetheless, the role of EFHD1, AHCYL1, and ALDH9A in atherosclerotic plaque remains underexplored in this specific context. These DEGs suggest potential predictive or regulatory functions in the progression of AS. Notably, EFHD1 was identified as the central gene through WGCNA conducted on symptomatic carotid plaques.

Research concerning EFHD1, a member of the EF-hand domain family known as D1, has been relatively limited. This 27 kDa mitochondrial protein contains two EF-hand domains that bind calcium (Ca^2+^) [68]. It plays a pivotal role in regulating mitochondrial metabolism, particularly at the intersection of ROS and calcium signaling. EFHD1 has been identified as a Ca^2+^ sensor that activates mitoflashes [69]. However, the role of EFHD1 as a regulator of mitochondrial metabolism remains underexplored. Furthermore, a recent study demonstrated that EFHD1 can influence the morphology and energy metabolism of mitochondria in dorsal root ganglion neurons [70]. These findings underscore the significance of EFHD1 in mitochondrial metabolism. Additionally, EFHD1 has been recognized as a potential prognostic marker in various tumor types, including gastric and colorectal cancer, as well as hepatocellular and clear cell renal cell carcinoma [71-74]. In our study, we observed a reduction in EFHD1 levels within AS plaques, as well as in macrophages exposed to ox-LDL. Therefore, EFHD1 may serve as a valuable target for both the diagnosis and treatment of AS.

To enhance our understanding of the role of immune cells in AS, we conducted a comprehensive assessment of immune cell infiltration using ssGSEA. It is well established that atherosclerotic lesions are characterized by significant infiltration of macrophages and T-cells. Interleukin-15 (IL-15) is a cytokine that shares numerous biological functions with IL-2, despite lacking sequence homology. IL-15 interacts with a heterotrimeric receptor composed of the β- and γ-subunits of the IL-2 receptor, along with its unique α-chain, designated as IL-15R-α [75, 76]. In our analysis of immune infiltration, we found that EFHD1 was negatively associated with MDSC, activated B cells, and natural killer cells. Interleukin (IL)-15 is a ligand that activates human NK cells through components of the IL-2 receptor, exhibiting a pattern similar, yet not identical, to that of IL-2 [77]. As demonstrated in the study by Carson *et al.*, IL-15 serves as a novel cytokine that activates human NK cells *via* components of the IL-2 receptor [78]. Both age and IL-15 play crucial roles in the pathogenesis of atherosclerosis. Research conducted by Tarantino *et al.* indicates that age and interleukin-15 levels are independently associated with intima- media thickness in patients with obesity-related non-alcoholic fatty liver disease (NAFLD). Additionally, a separate study demonstrated that IL-15 polymorphisms are associated with an increased risk of developing subclinical atherosclerosis and are linked to metabolic syndrome, including central obesity and fatty liver [79]. IL-15 is found to be upregulated in atherosclerotic lesions in both humans and animals [80], and it may play a role in the recruitment and activation of T cells during atherogenesis. Furthermore, IL-15 may facilitate antigen-independent T-cell activation within atherosclerotic plaques [81]. Research has shown that EFHD1 expression correlates with tumor-infiltrating neutrophils and can serve as a prognostic indicator in gastric cancer [74]. Moreover, EFHD1 is known to regulate metabolic changes and the development of pro/pre B cells [82], indicating its potential role in the immune microenvironment of AS. EFHD1 appears to influence the onset and progression of inflammation through various mechanisms. Notably, following the silencing of EFHD1, a decrease in IL-1β and TNF-α levels was observed, which is consistent with previous findings [83]. The initiation of inflammatory processes and the accumulation of lipid-laden macrophages are key indicators of atherosclerosis. Inflammation occurs locally within blood vessels as lipids and cholesterol accumulate, accompanied by an increase in inflammatory factors in the bloodstream. In regions of vascular damage, there is an upregulation of adhesion molecules, including vascular cell adhesion molecule-1 (VCAM-1), intracellular adhesion molecule-1 (ICAM-1), and E-Selectin. Macrophages engulf ox-LDL and transform into lipid-laden foam cells, which subsequently release inflammatory mediators such as tumor necrosis factor-α (TNF-α) and interleukin-6 (IL-6) [61]. The presence of these adhesion molecules and inflammatory mediators can activate the JAK/STAT signaling pathways. This activation results in damage to the vascular structure and the formation of fatty streaks within the vascular wall, leading to decreased elasticity and ultimately contributing to the development of atherosclerotic plaques. Furthermore, previous investigations suggest that the JAK/STAT pathway plays a crucial role in modulating the inflammatory response in the arterial adventitia, influencing both the promotion of atherogenesis and the inhibition of atheroprotective mechanisms [84]. The use of JAK/STAT inhibitors has shown potential in reducing cardiovascular risk. Our research indicates that the removal of EFHD1 results in elevated levels of the proteins JAK2 and STAT3, highlighting a potential new target within the JAK/STAT pathway for modulating inflammatory responses in plaques.

In the group with low EFHD1 levels, GSEA results indicated significant enrichment of the cAMP and calcium signaling pathways, cytokine-cytokine receptor interactions, and neuroactive ligand-receptor interactions. Calcium ions function as second messengers within cells, playing a crucial role in the regulation of cellular functions. EFHD1 directly interacts with the mitochondrial calcium uniporter (MCU) and inhibits mitochondrial Ca^2+^ uptake by blocking MCU activity. In our study, silencing EFHD1 did not result in significant changes in mitochondrial or cytoplasmic calcium ion levels. Similarly, Hou *et al*. reported that EFHD1 knockdown did not lead to significant alterations in mitochondrial and cytosolic Ca^2+^ transients [69]. Additionally, we observed decreased ATP and ROS levels, alongside an upregulation of glycolysis-related metabolic enzymes, including ALODA and PKM2, following EFHD1 knockdown. Vulnerable plaques, also known as thin fibrous cap atheromata, are classified as “high risk” due to their susceptibility to rupture and thrombosis. These plaques exhibit a significant presence of inflammatory cells, including macrophages, T cells, and a limited number of smooth muscle cells [85]. Macrophages are believed to play a pivotal role in plaque calcification. A transition in macrophage phenotype-from M1-like to M2-like-can occur through metabolic reprogramming, as the metabolic conditions of macrophages influence their functional characteristics. Importantly, M2-like macrophages heavily depend on the mitochondrial oxidative phosphorylation (OXPHOS) pathway [86], whereas M1-like macrophages tend to favor anaerobic glycolysis [87]. Our findings indicate that EFHD1 in macrophages may also influence their functional phenotype by impacting mitochondrial energy metabolism. This, in turn, has implications for plaque stability, necessitating further investigation into the underlying mechanisms.

Furthermore, our research identified a correlation between EFHD1 expression and MMP-9 levels, revealing that EFHD1 knockdown leads to increased MMP-9 production. Within the core of atherosclerotic plaques, matrix metalloproteinases (MMPs) are produced by large macrophages and macrophage-derived foam cells, which compromise the integrity of the fibrous cap, resulting in plaque instability and potential rupture. These MMPs can degrade the extracellular matrix and the basement membrane, thereby facilitating the migration of macrophages and vascular smooth muscle cells within the intima. The MMP family, particularly MMP-1, MMP-2, and MMP-9, is associated with unstable plaques due to their capacity to degrade extracellular matrix components [88]. The levels of MMPs may be correlated with EFHD1 expression and could serve as indicators of the progression of vulnerable plaques. Our findings suggest that EFHD1 may act as a novel biomarker and therapeutic target for mitigating the progression and vulnerability of atherosclerotic disease.

Moreover, this study is the first to construct the lncRNA-miRNA-EFHD1 regulatory network in the context of AS plaque formation. We identified a total of 35 regulatory miRNAs alongside 75 lncRNAs. Both lncRNAs and miRNAs serve as crucial regulators in various biological processes, with their dysregulated expression significantly associated with the onset and progression of several diseases. Within this framework, miR-149 and miR-491 have been shown to influence atherosclerosis by modulating autophagy, apoptosis, and inflammatory responses [89, 90]. Studies indicate that lncRNA MALAT1 interacts with miR-330-5p, activating the NF-κB signaling pathway in ox-LDL-treated macrophages [90, 91]. Furthermore, lncRNA XIST has been identified as a promoter of atherosclerosis by regulating the miR-599/TLR4 pathway or the miR-221-3p/TRAF6 pathway [92, 93]. Additionally, lncRNA SNHG16 has been observed to facilitate atherosclerosis and enhance vascular smooth muscle cell proliferation through the miRNA-22-3p/high-mobility group box 2 (HMGB2) axis [94]. Regarding other less frequently discussed miRNAs and lncRNAs, further investigations are required to elucidate their molecular mechanisms in AS.

The current study has several limitations. Firstly, the data utilized for our investigation was sourced from a publicly available repository and was restricted to a single dataset, primarily due to the limited availability of AS datasets. This limitation resulted in the exclusion of significant clinical details, thereby impeding our ability to conduct comprehensive prognostic evaluations. Secondly, while we examined the mechanism by which EFHD1 influences macrophage function at the cellular level, further validation through animal studies is crucial to elucidate the role of EFHD1 in the formation of AS plaques. Lastly, the acquisition of clinical samples is essential for a comprehensive understanding of the diagnostic and therapeutic implications of EFHD1. Should clinical plaque tissue be obtained in the future, it will be further validated through experiments to provide additional evidence for our study.

## CONCLUSION

We identified seven hub genes (ALDH3A2, UCP1, BCL2, EFHD1, AHCYL1, HTRA2, and ALDH9A1) associated with mitochondrial function and aging from the GEO database in both asymptomatic and symptomatic carotid plaques. Notably, EFHD1 was found as the final hub gene and validated through GSE163154, GSE43292, GSE104140, GSE28829, GSE120521, and GSE41571 databases. Besides, EFHD1 expression was found to be decreased in atherosclerotic tissues and ox-LDL-treated macrophages. Knockdown of EFHD1 reduced levels of pro-inflammatory cytokines, including IL-1β and TNF-α, as well as regulated the JAK2/STAT3 pathway and mitochondrial energy metabolism. Therefore, EFHD1 may affect inflammation and mitochondrial energy metabolism through the calcium signaling pathway. This discovery has the potential to enhance early diagnosis and improve treatment strategies for AS.

## Figures and Tables

**Fig. (1):**
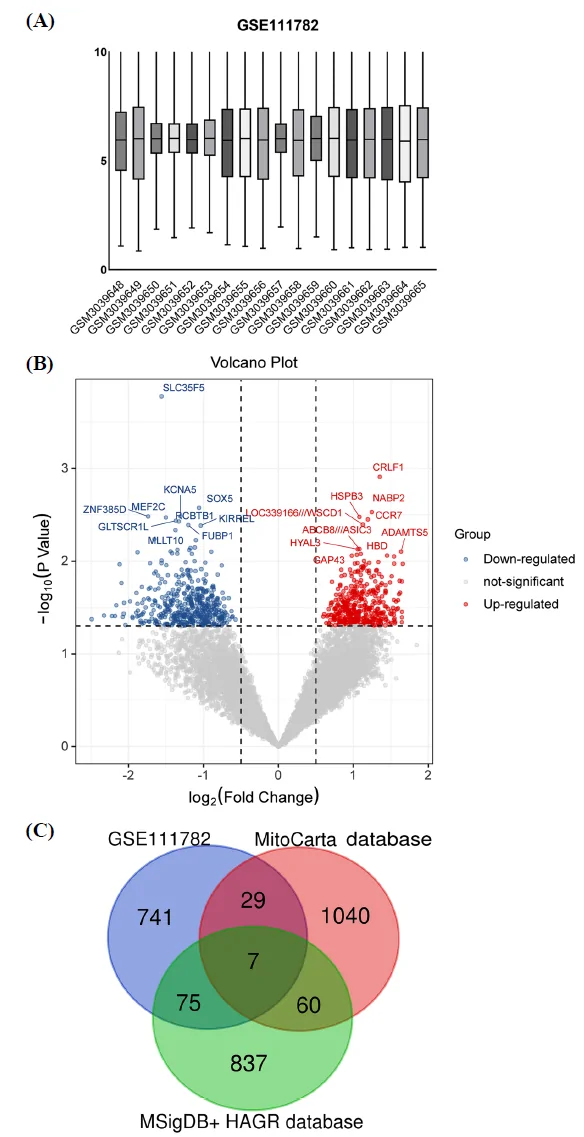
Data preprocessing for DEGs. (**A**) Box plots of raw data normalized between samples. (**B**) Volcano plot of DEGs. Blue dots indicate statistically significant downregulated genes and red dots indicate statistically significant upregulated genes. (**C**) Venn diagram of overlapping genes in GSE111782 and mitochondrial and aging-related genes.

**Fig. (2):**
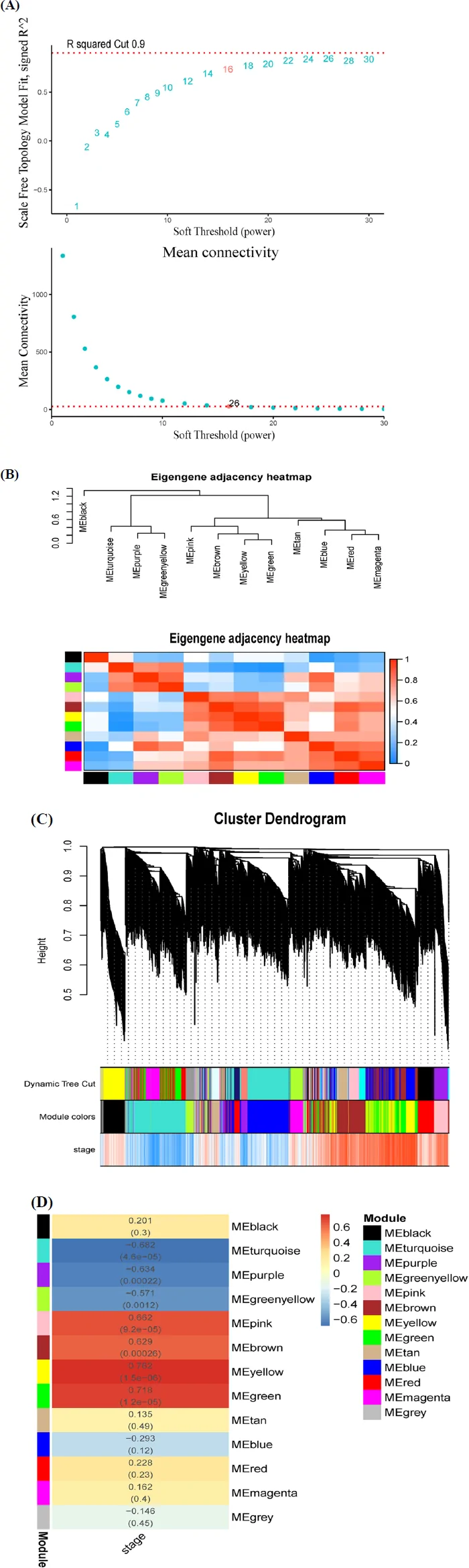
Identification of the highly correlated gene modules in WGCNA. (**A**, **B**) The Clusterdendrogram inin the GSE28829 and GSE104140. (**C**, **D**) Heatmap illustrating the module-trait relationships in the GSE28829 and GSE104140.

**Fig. (3):**
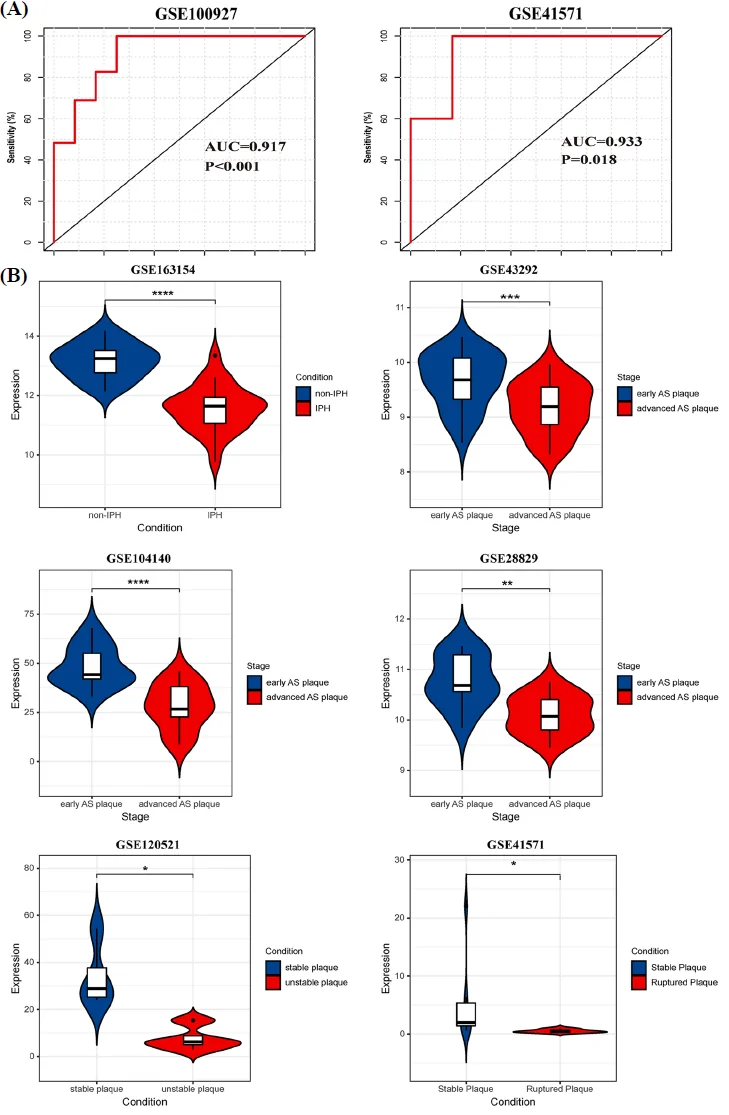
The expression levels and diagnostic efficacy of EFHD1 in AS datasets. (**A**) The ROC curves showing AUC values of EFHD1 in GSE100927 and GSE41571. (**B**) EFHD1 expression levels in six As databases, with blue violin plots representing controls, red representing unstable/advanced plaque/IPH. Students’t-test with *p* < 0.05 was used to determine statistical significance. (*p < 0.05;**p < 0.01;***p <0.001;****p < 0.0001). AS, atherosclerosis; AUC, area under curve.

**Fig. (4):**
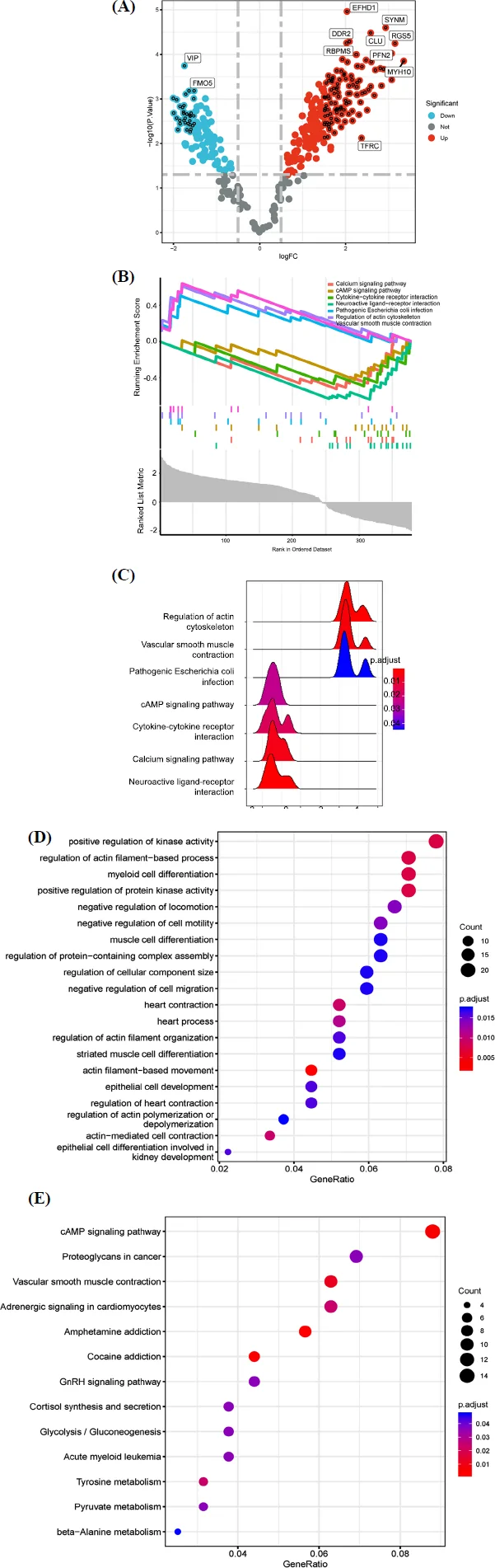
GSEA, GO and KEGG functional analysis of EFHD1. (**A**) The volcano map of GSE111782 after regrouping according to the levels of EFHD1 expression. (**B**, **C**) GSEA analysis of hub genes. (**D**, **E**) The enrichment analysis results of GO and KEGG pathway.

**Fig. (5):**
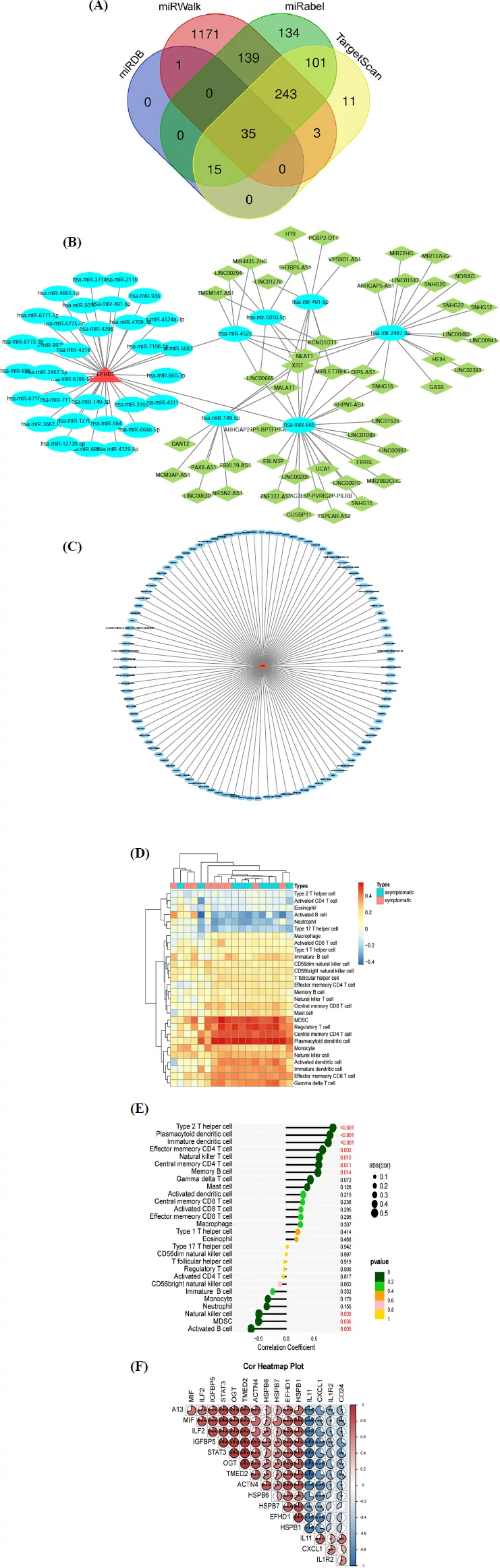
Prediction of lncRNA-miRNA-EFHD1 regulatory network and chemical network of EFHD1. (**A**) Venn diagram of overlapping miRNAs in four online miRNA databases. (**B**) The lncRNA-miRNA-EFHD1 network included EFHD1(red triangle), 35 regulatory miRNAs (blue ellipse nodes),and 75 regulatory lncRNAs (green diamond nodes). (**C**) The drug network of EFHD1 using the Comparative Toxicogenomics Database. (**D**) The heat map of immune infiltration. Each row represents one sample, either early or advanced plaque, each column represents a type of immune cell or immune function. (**E**) Correlation analysis between immune infiltration and EFHD1. (**F**) The correlation heatmap of immune-associated genes of EFHD1.

**Fig. (6):**
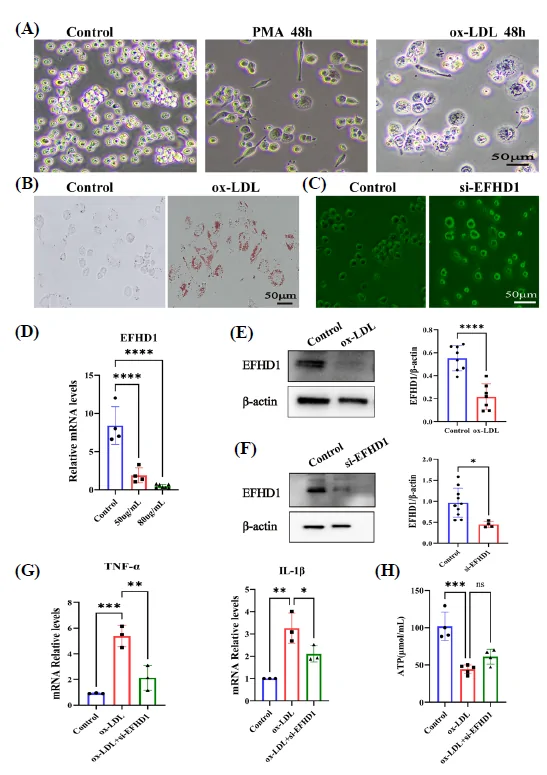
The expression of EFHD1 decreased in the foam cell. (**A**) The morphological changes in THP-1 cells after stimulating with PMA and ox-LDL, scale bar: 50 μm. (**B**) Oil red O staining of the THP-1 cells, scale bar: 50 μm. (**C**) The immunofluorescence staining after si-EFHD1 transfection. (**D**) Relative mRNA levels of EFHD1 in 50μg/ml and 80μg/ml ox-LDL-treated groups. (**E**) Western blot of EFHD1 expression in ox-LDL-treated group and control group. (**F**) Western blot of EFHD1 expression in EFHD1-knockdown group and control group. (**G**) Difference in relative mRNA levels of TNF-α and IL-1β among control,ox-LDL and ox-LDL +si-EFHD1 groups. (**H**) The ATP production among control,ox-LDL and ox-LDL +si-EFHD1 groups. Full-length blots/gels are presented in Supplementary Fig. (**1**).

**Fig. (7):**
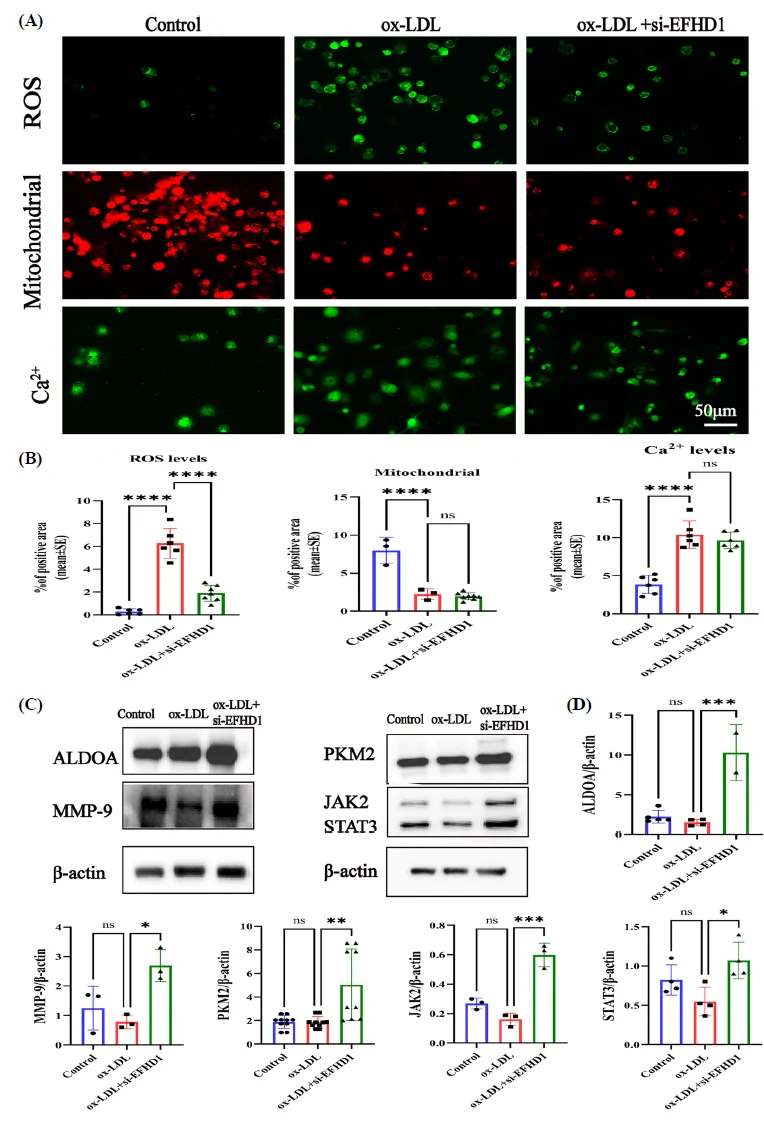
Effect of EFHD1 knockdown on the expression of AS-relevant factors. (**A**) Immunofluorescence staining of ROS,mitochondrial and Ca^2+^ concentration, scale bar: 50 μm. (**B**) Relative statistical results of the stained area in A. (**C**) Difference in proteins expression among control,ox-LDL and si-EFHD1 groups. (**D**) Relative statistical results of the protein bands in C. Full-length blots/gels are presented in Supplementary Fig. (**1**). (*p < 0.05, **p < 0.01, ***p < 0.001, ****p < 0.0001).

**Fig. (8):**
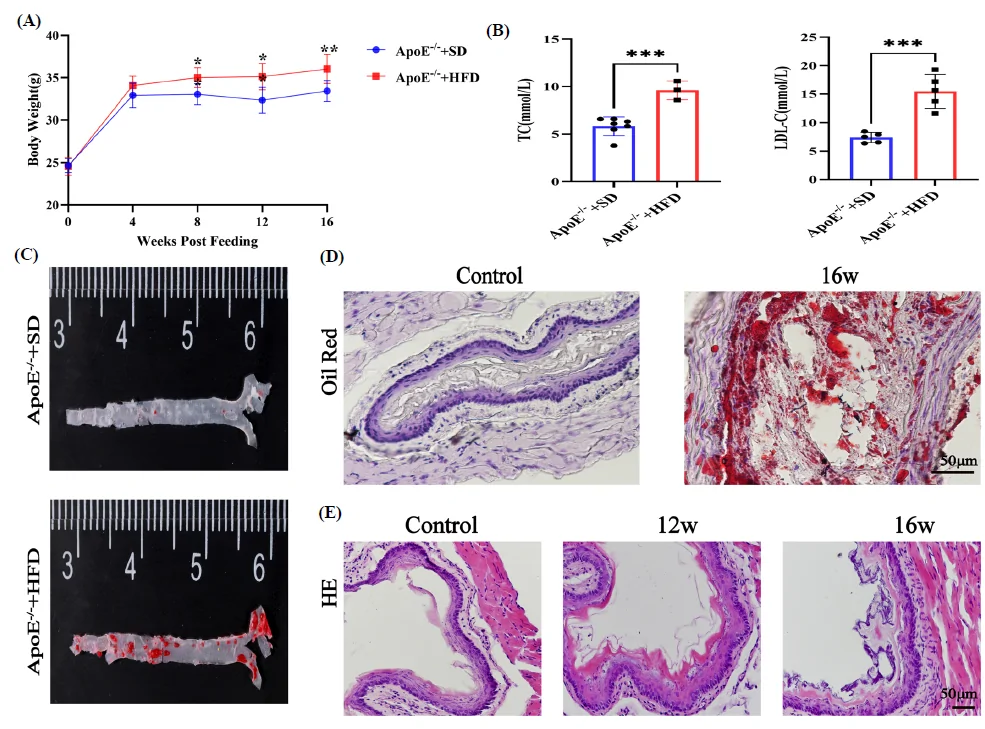
The AS animal model. (**A**) The body weights of the ApoE^-/-^ mice with HFD and with SD.*p < 0.05, **p < 0.01, ***p < 0.001, ****p < 0.0001. (**B**) The circulating TC and LDL-C levels in the AS model and control group. (**C**, **D**) Oil red O staining of the mouse aortic tissues. (**E**) HE staining of aortic tissues treated with HFD or SD *in vitro*. Scale bar: 50 μm. **Abbreviations:** SD:standard diet; HFD:high-fat diet.

**Fig. (9):**
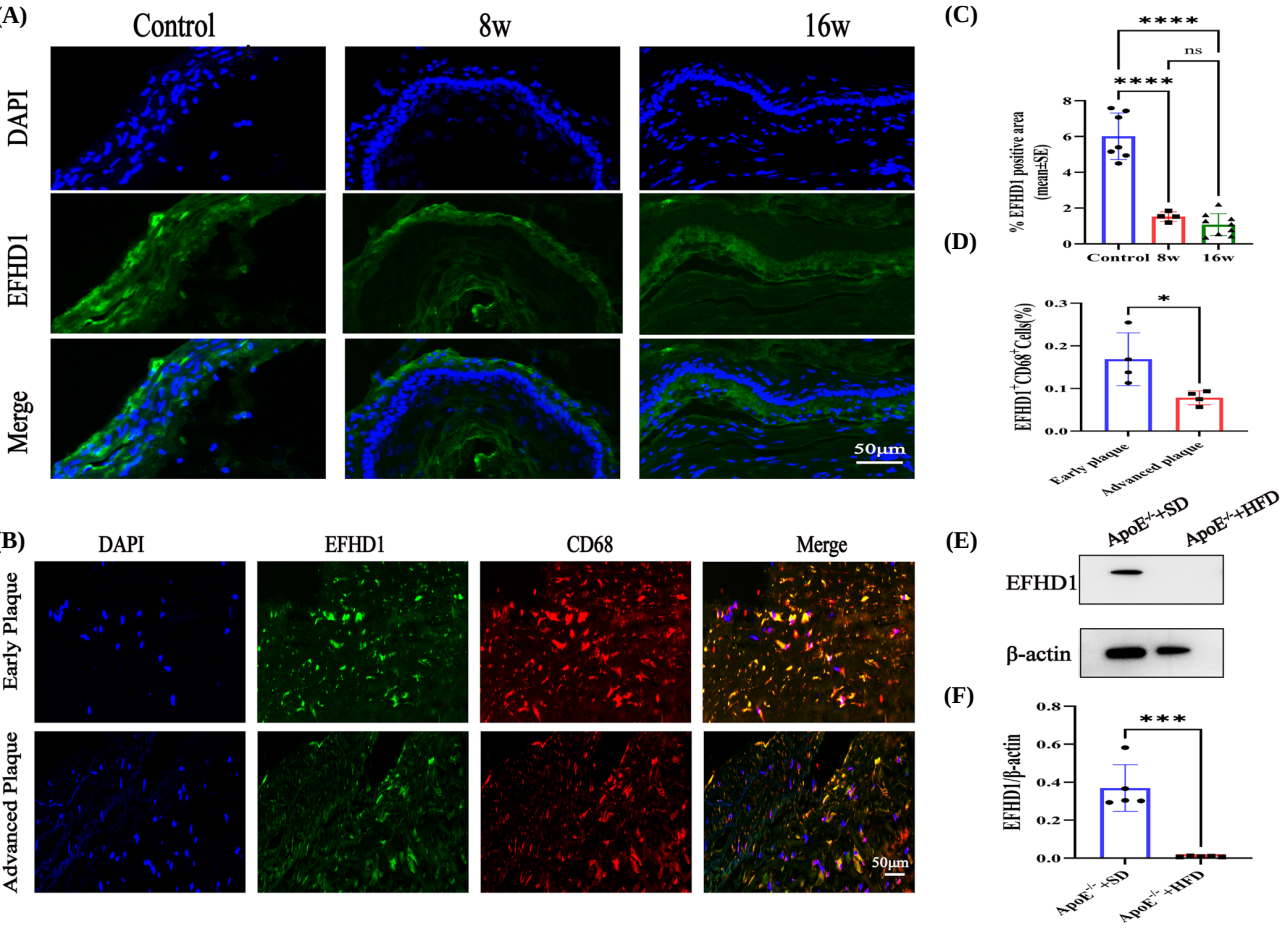
Animal subject validation. (**A, B**) Immunofluorescence staining of EFHD1 and CD68 in aortic tissues, scale bar: 50 μm. Values are mean ± SD. (**C**, **D**) Statistical differences were determined by two-tailed unpaired Student's t-test. (**E**, **F**) Western blot of EFHD1 expression in aortic tissues. Full-length blots/gels are presented in Supplementary Fig. (**1**). *p < 0.05, **p < 0.01, ***p < 0.001, ****p < 0.0001.

**Table 1:** Clinical factors related to the expression of EFHD1 in the GEO database.

**Dataset**	**Factors**	**B**	**Sig.**	**OR**	**95%CI**
GSE43292	Carotid atheroma	-2.053	0.001	0.128	0.039-0.422
GSE104140	Advanced atherosclerosis	-0.232	0.011	0.793	0.663-0.949
GSE163154	IPH	-3.311	0.001	0.036	0.005-0.267
GSE28829	Advanced atherosclerosis	-3.512	0.009	0.03	0.002-0.418

## Data Availability

Data was downloaded from the public database online at the GEO database under accession numbers GSE111782, GSE28829, GSE104140, GSE100927, GSE41571, GSE163154, GSE43292, and GSE120521.

## LIST OF ABBREVIATIONS

AS: Atherosclerosis
GEO: Gene Expression Omnibus
MSigDB: MitoCarta, Molecular Signatures Database
HAGR: Human Aging Genomic Resources
EFHD1: EF-hand domain family member D1
ROC: Receiver Operating Characteristic
Ox-LDL: Oxidized Low-density Lipoprotein
MDSC: Myeloid Suppressor Cells
ROS: Reactive Oxygen Species
