# Supplementary material for: EFHD1: A Potential Prognostic Biomarker Related to Mitochondrial Function and Aging in Atherosclerosis Plaque
Source: Curr Med Chem. 2025 Jul 21;33(25):5373–99. doi: 10.2174/0109298673356904250628182630 (PMC13692762; doi:10.2174/0109298673356904250628182630)
Supplement: Supplementary file 1 [file CMC-33-25-5373_SD1.pdf]

Supplementary Material

EFHD1: A Potential Prognostic Biomarker Related to Mitochondrial Function and Aging in Atherosclerosis Plaque

Lin Wang[1,#], Yuxiu Han[2,#], Yu Qiao[1,#], Tao Yan[1,*], Zhi Qi[1], Wei Zhang[1], Ling Xin[1], Mingjing Yu[1] and Zhili Chen[1]

[1]Key Laboratory of Post-Neurotrauma, Neurorepair, and Regeneration in Central Nervous System, Ministry of Education and Tianjin City, Department of Neurology, Tianjin Neurological Institute, Tianjin Medical University General Hospital, Tianjin, 300052, China; [2]Department of Neurology, Tianjin Hospital, Tianjin University, Tianjin, 300211, China

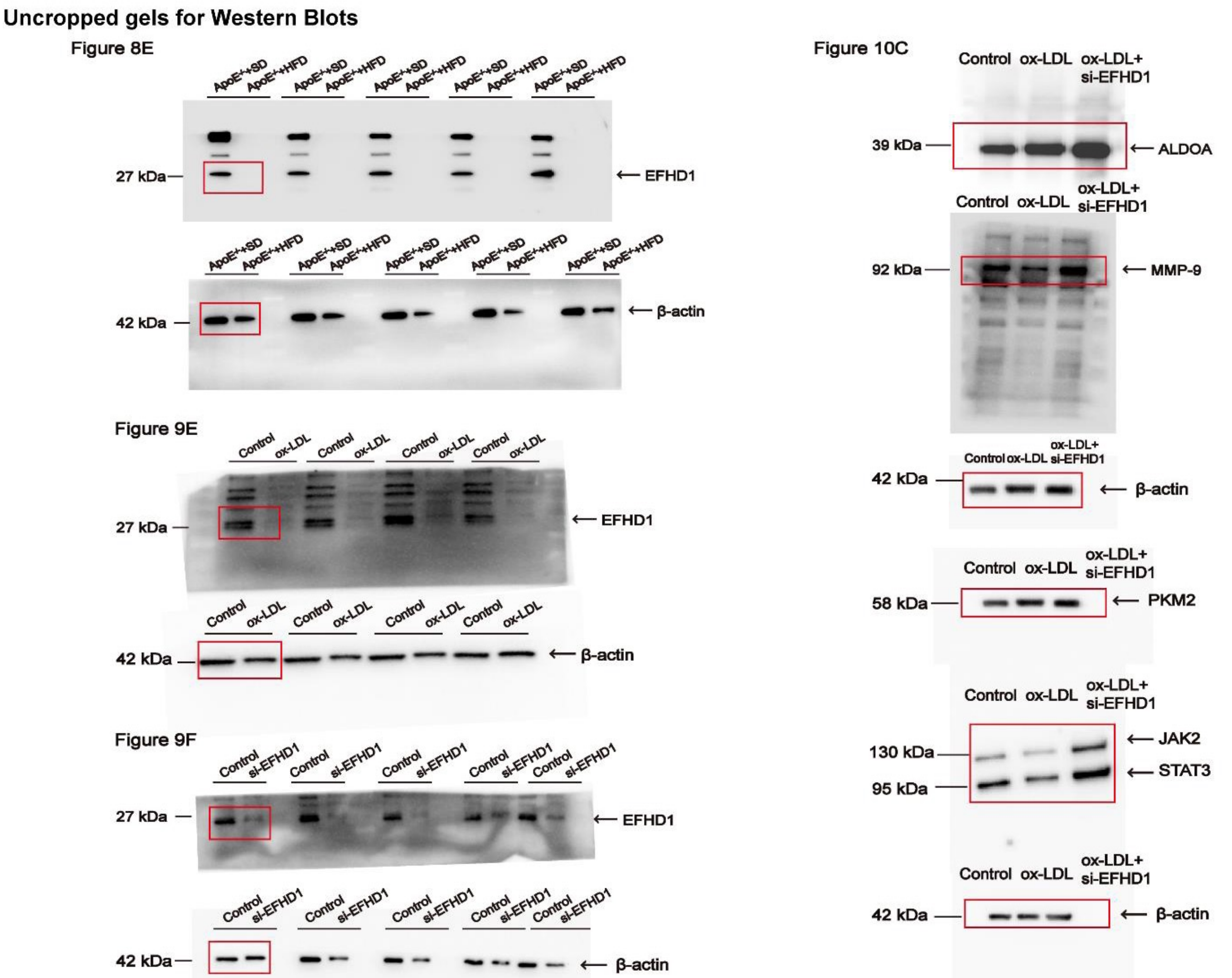

Fig. (S1). The full uncropped gels for Western Blots was presented in supplementary Fig. (**1**).
